# Comparison of efficacy and safety between simultaneous integrated boost intensity-modulated radiotherapy and standard-dose intensity-modulated radiotherapy in locally advanced esophageal squamous cell carcinoma: a retrospective study

**DOI:** 10.1007/s00066-021-01894-y

**Published:** 2022-01-14

**Authors:** Wang Lan, Liu Lihong, Han Chun, Liu Shutang, Wang Qi, Xu Liang, Li Xiaoning, Liu Likun

**Affiliations:** Department of Radiation Oncology, The Fourth Hospital of Hebei Medical University, 050011 Shijiazhuang, China

**Keywords:** Esophageal neoplasms, Radiotherapy, Fractionation, Simultaneous integrated boost intensity-modulated radiotherapy, Survival

## Abstract

**Objective:**

This study aimed to evaluate the efficacy and safety of simultaneous integrated boost intensity-modulated radiotherapy (SIB-IMRT) versus standard-dose intensity-modulated radiotherapy (SD-IMRT) in the treatment of locally advanced esophageal squamous cell carcinoma.

**Methods:**

From July 2003 to March 2014, 1748 patients in a single center who received definitive chemoradiotherapy were included in the analysis. A total of 109 patients who underwent SIB-IMRT and fulfilled all inclusion and exclusion criteria were identified as the study group. A total of 266 patients who underwent SD-IMRT (60 Gy/30 fractions, 2 Gy/fraction, 1 time/day, 5 times/week) during the same period were selected as the control group. Propensity score matching (PSM) was used to balance the baseline characteristics. Survival status, treatment failure mode, and the occurrence of adverse events were compared between the two groups.

**Results:**

There were more women and more cervical and upper thoracic cancers (*P* = 0.038, < 0.001, respectively) in the SIB-IMRT group before case matching. The median progression-free survival (PFS) in the SD-IMRT and SIB-IMRT groups was 22 and 19 months, respectively, and the median overall survival duration was 24 and 22 months, respectively, with χ^2^ = 0.244 and *P* = 0.621. After PSM of 1:1, 138 patients entered the final analysis (69 cases from each group). The median PFS of the SD-IMRT group and the SIB-IMRT group was 13 and 18 months, respectively, with χ^2^ = 8.776 and *P* = 0.003. The 1‑, 3‑, and 5‑year overall survival rates were 66.7, 21.7, and 8.7% and 65.2, 36.2, and 27.3%, respectively, and the median overall survival duration was 16 and 22 months, respectively, with χ^2^ = 5.362 and *P* = 0.021. Treatment failure mode: 5‑year local regional recurrence rates of SD-IMRT and SIB-IMRT were 50.7 and 36.2%, respectively, with χ^2^ = 2.949 and *P* = 0.086. The 5‑year distant metastasis rates of the two groups were 36.2 and 24.6%, respectively, with χ^2^ = 2.190 and *P* = 0.139. Adverse events: 3 patients experienced grade 4–5 toxicity (2.2%), including one case of grade 4 radiation esophagitis and two cases of grade 5 radiation pneumonitis, all in the SD-IMRT group; 14 patients experienced grade 3 adverse events (10.1%), primarily including radiation esophagitis, radiation pneumonitis, and hematological toxicity.

**Conclusion:**

The technique of SIB-IMRT was safe and reliable compared with SD-IMRT. In addition, SIB-IMRT had locoregional control advantages and potential survival benefits.

**Supplementary Information:**

The online version of this article (10.1007/s00066-021-01894-y) contains supplementary material, which is available to authorized users.

Simultaneous integrated boost intensity-modulated radiotherapy (SIB-IMRT) is an advantageous radiotherapy technique that can offer the unique capability of dose escalation by means of a simultaneous integrated boost (SIB). It can offer the advantage of delivering a higher dose to the primary tumor while conventional lower doses are used simultaneously to treat subclinical lesions or elective node regions. In simultaneous boosting, the total number of radiation therapy (RT) fractions is kept constant. SIB-IMRT has been successfully implemented to treat cancers of various regions such as the head and neck [1–4], prostate [5], and rectum [6, 7]. In the field of esophageal cancer, SIB-IMRT technology also has application potential. However, because the esophagus is a lumen organ and adjacent to blood vessels, based on the risk of perforation and bleeding during the application of SIB-IMRT, the dose escalation range from the clinical target volume (CTV) to the gross tumor volume (GTV) is relatively small. Based on the research results of several phase I/II clinical trials [8–11], it is safe and feasible to use SIB-IMRT technology to escalate the total dose to a primary tumor to 59.92–70 Gy, with a single fraction dose of 2.14–2.8 Gy. However, whether this technology could provide final local control or a survival benefit in the treatment of esophageal cancer still lacks sufficient research data [12–14]. Based on the aforementioned background, this study retrospectively analyzes a large number of esophageal cancer cases treated in a single center of our hospital. Patients who received SIB-IMRT were selected as the study group, and the control group was set using 1:1 propensity score matching (PSM). The purpose was to explore whether dose escalation using SIB-IMRT technology might be beneficial in certain esophageal cancer patients.

## Materials and methods

### Patients and eligibility criteria

**Fig. 1:**
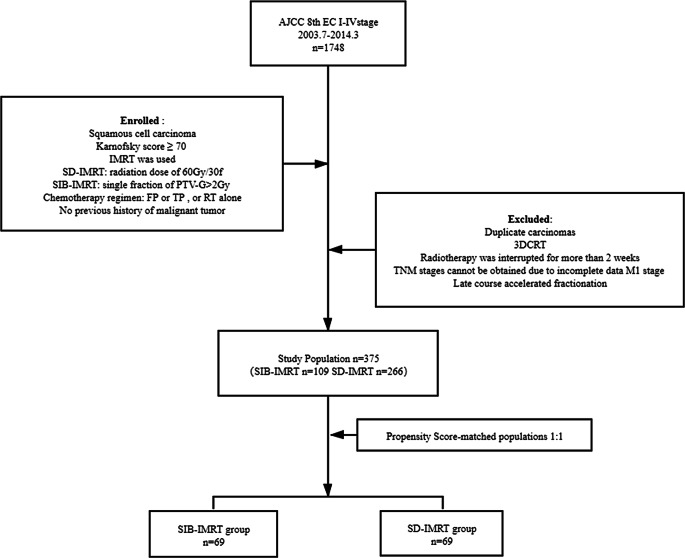
Study flow diagram. *FP* fluorouracil and cisplatin, *TP* paclitaxel and cisplatin

From July 2003 to March 2014, 1748 patients with esophageal cancer treated by definitive radiotherapy in our hospital were analyzed. The inclusion criteria consisted of the following: 1) squamous cell carcinoma confirmed by pathology; 2) a Karnofsky score ≥ 70; 3) intensity-modulated radiotherapy technology was used; 4) for patients undergoing SIB-IMRT, the single fraction dose for the planning target volume (PTV) of the GTV region was > 2 Gy, for those who received conventionally fractionated radiotherapy, the 60 Gy/30 fractions mode was selected as the standard-dose intensity-modulated radiotherapy (SD-IMRT) for the control; 5) chemotherapy consisting of the FP (5-fluorouracil + cisplatin) or TP (paclitaxel + cisplatin) regimen was used or radiotherapy alone; and 6) no history of malignant tumor. The exclusion criteria consisted of the following: 1) multi-primary esophageal carcinoma; 2) conformal radiotherapy; 3) radiotherapy interruption for more than 2 weeks; 4) insufficient imaging data and unable to define the TNM stage; 5) M1 stage patients; and 6) late-course accelerated hyperfractionated radiotherapy. After screening according to the inclusion and exclusion criteria, 375 cases met the enrollment conditions with 109 cases in the SIB-IMRT group and 266 cases in the SD-IMRT group. The study flow diagram is shown in Fig. 1.The clinical data and comparability tests of the two groups are shown in Table 1. The staging was based on the eighth edition of the American Joint Committee on Cancer (AJCC) clinical TNM (cTNM) staging standard for esophageal cancer.

**Table 1:** Patient characteristics before and after PSM

Characteristics	Before PSM (*n* = 375)			After PSM (*n* = 138)		
	SD-IMRT group (*n* = 266)	SIB-IMRT group (*n* = 109)	*P*-value^a^	SD-IMRT group (*n* = 69)	SIB-IMRT group (*n* = 69)	*P* -value^a^
*Sex (n)*						
Male	179 (67.3%)	61 (56.0%)	0.038*	43 (62.3%)	43 (62.3%)	1.000
Female	87 (32.7%)	48 (44.0%)		26 (37.7%)	26 (37.7%)	
*Age (years)*						
≤ 70	177 (66.5%)	91 (83.5%)	0.095	49 (71.0%)	56 (81.2%)	0.741
> 70	89 (33.5%)	18 (16.5%)		20 (29.0%)	13 (18.8%)	
Median (range)	65 (41 ~ 86)	64 (39 ~ 80)		65 (41 ~ 84)	64 (39 ~ 80)	
*Lesion length (by barium meal, cm)*						
≤ 5.5 cm	135 (50.8%)	67 (61.5%)	0.459	35 (50.7%)	43 (62.3%)	0.158
> 5.5 cm	131 (49.2%)	42 (48.5%)		34 (49.3%)	26 (37.7%)	
Median (range)	5.6 (0 ~ 13.9)	5.5 (2.4 ~ 10.2)		5.3 (0–10.2)	5.4 (2.4 ~ 10.2)	
*Tumor site (n)*						
Cervical	8 (3.0%)	17 (15.6%)	< 0.001*	6 (8.7%)	7 (10.1%)	0.469
Upper	79 (29.7%)	42 (38.5%)		20 (29.0%)	25 (36.2%)	
Middle	143 (53.8%)	42 (38.5%)		38 (55.1%)	29 (42.0%)	
Lower	36 (13.5%)	8 (7.4%)		5 (7.2%)	8 (11.6%)	
*T stage (n)*						
T_1+2_	41 (15.4%)	25 (22.9%)	0.221	12 (17.4%)	14 (20.3%)	0.904
T_3_	57 (21.4%)	21 (19.3%)		14 (20.3%)	14 (20.3%)	
T_4_	168 (63.2%)	63 (57.8%)		43 (62.3%)	41 (59.4%)	
*N stage (n)*						
N_0_	22 (8.3%)	15 (13.8%)	0.156	3 (4.3%)	9 (13.0%)	0.180
N ^+^	244 (91.7%)	94 (86.2%)		66 (95.7%)	60 (87.0%)	
*GTV volume (cm*^*3*^*)*						
≤ 45 cm^3^	125 (47.0%)	61 (56.0%)	0.054	34 (49.3%)	34 (49.3%)	0.875
> 45 cm^3^	141 (53.0%)	48 (44.0%)		35 (50.7%)	35 (50.7%)	
Median (range)	47.9 (1.8 ~ 189.2)	41.2 (3.8 ~ 174.7)		46.3 (7.9 ~ 171.5)	45.2 (8.4 ~ 174.7)	
*Treatment regimen*						
*RT alone*	118 (44.4%)	59 (54.1%)	0.119	35 (50.7%)	32 (46.4%)	0.902
*Sequential Chemoradiotherapy*	54 (20.3%)	23 (21.1%)		14 (20.3%)	15 (21.7%)	
*CCRT*	94 (35.3%)	27 (24.8%)		20 (29.0%)	22 (31.9%)	
*Prescription dose (Gy)*^b^						
Range	60	59.92 ~ 66/50.4 ~ 60.00 ^b^	–	60		59.92 ~ 66.00/50.4 ~ 60.00 ^b^
Median	60	63/57	–	60	64/59	–
*Fractions*						
Range	30	27 ~ 31	–	30	27 ~ 31	–
Median	30	30	–	30	30	–

*PSM* propensity score matching, *PTV‑G* the planning target volume of GTV‑p and GTV‑n, *PTV‑C* the planning tartget volume of CTV

^a^χ^2^ or two-independent-sample tests

^b^Before PSM: the prescription dose range of PTV_-G_ was 59.92–66.00 Gy, median: 63.00 Gy; the prescription dose range of PTV_-C_ was 50.4–60.00 Gy, median: 57.00 Gy. After PSM: the prescription dose range of PTV_-G_ was 59.92–66.00 Gy, median: 64 Gy; the prescription dose range of PTV_-C_ was 50.4–60.00 Gy, median: 59 Gy

*Statistically significant *p*-value

### Radiation therapy

**Fig. 2:**
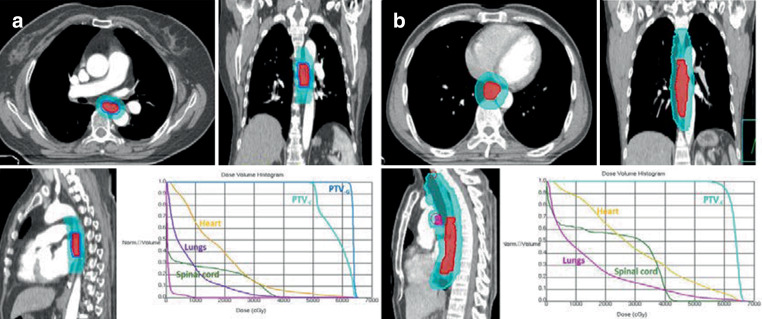
The representative SIB-IMRT and SD-IMRT planning images with contours and the dose–volume histogram. **a** Target contour of SIB-IMRT plan for a patient with ESCC, displayed on the axial, coronal, and sagittal planes through the primary tumor. Dose–volume histograms for the relevant structures. *Navy blue shading* indicates the PTV_-G_ (PTV of primary tumor and involved nodes, with the prescription dose of 63 Gy/28 fractions), *light blue shading* indicates the PTV_-C_ (PTV of clinical target volume, with the prescription dose of 50.4 Gy/28 fractions). **b** Target contour of SD-IMRT plan for a patient with ESCC, displayed on the axial, coronal, and sagittal planes through the primary tumor. Dose–volume histograms for the relevant structures. *Light blue shading* indicates the PTV_-C_ (PTV of clinical target volume, with the prescription dose of 60 Gy/30 fractions)

All patients underwent computed tomography (CT)-based treatment simulation in the supine position, and 3‑mm thick images were obtained throughout the entire neck, thorax, and upper abdomen. The scanned images were transferred to a three-dimensional (3D) planning system. The GTV, CTV, PTV, and normal organs at risk (OAR) were delineated layer by layer. The GTV included primary tumors (GTV_-P_) and lymph node metastasis (GTV_-n_). The GTV_-P_ included all esophageal tumors that were found using a CT scan, esophageal barium, endoscopy, endoscopic ultrasonography (EUS), and PET-CT. The GTV_-n_ was defined as any lymph node diagnosed as or highly suspected of being metastatic. The CTV of a primary tumor (CTV_-P_) was defined as the GTV_-P_ plus a 2-cm margin superiorly and inferiorly and a 0.5-cm margin laterally along the esophagus. For the CTV of the lymph node (CTV_-n_), involved-field radiotherapy (IFI) was used for the majority of patients. However, if the primary tumor was in the cervical or upper thoracic esophagus, the CTV_-n_ encompassed the elective nodal area including the bilateral supraclavicular and upper mediastinal lymph node regions. The PTV of the clinical target volume (PTV_-C_) was generated by adding a 1-cm margin craniocaudally, a 0.5-cm margin laterally along the CTV_-P_, and a uniform 0.5-cm margin around CTV_-n_. The PTV_-G_ was defined using the GTV (GTV_-P_ + GTV_-n_) plus a 0.3–0.5 cm margin. For patients undergoing SIB-IMRT, the PTV_-G_ and PTV_-C_ received two prescription doses simultaneously. The lower dose was delivered to the PTV_-C_, and the higher dose was escalated to the PTV_-G_. For patients undergoing SD-IMRT, only one prescription dose was delivered to the PTV_-C_. A prescription dose was defined as 95% of the receiving dose of the PTV, with the difference of the internal target dose uniformity of < 5%, and the internal target maximum dose point of ≤ 110%. The OAR included the spinal cord, lungs, and the heart. The treatment plan generally required the entire lungs V5 ≤ 55–60%, V20 ≤ 25–30%, and V30 ≤ 18%; a mean heart dose of ≤ 26–30 Gy; and a maximum spinal cord dose of < 45 Gy. For the SIB-IMRT group, the prescribed doses were 50.4–60 Gy/27–31 fractions (1.8–2.0 Gy/fraction) to the PTV_-C_ and 59.92–66 Gy/27–31 fractions (2.06–2.29 Gy/fraction) to the PTV_-G_ (with an EQD2 of 60.38–67.66 Gy). The prescribed doses of the SD-IMRT group were 60 Gy/30 fractions, 2 Gy/fraction, 1 time/day, and 5 times/week. The representative SIB-IMRT and SD-IMRT planning images with contours and the dose–volume histogram are shown in Fig. 2.

### Chemotherapy

A total of 198 cases of the 375 patients received chemotherapy, including 54 cases of sequential chemoradiotherapy and 94 cases of concurrent chemoradiotherapy in the SD-IMRT group, 23 cases of sequential chemoradiotherapy, and 27 cases of concurrent chemoradiotherapy in the SIB-IMRT group. The chemotherapy regimen was FP or TP [15–18] with the following usage: cisplatin 75 mg/m^2^, divided into 3 to 5 days, 5‑FU 450–500 mg/m^2^ × 5 days (first to fifth days); or paclitaxel 135 mg/m^2^, the first day intravenously, with cisplatin 25 mg/m^2^ × 3 days (days 2, 3, and 4). Concurrent chemotherapy was given during the first and fourth or fifth weeks of radiotherapy.

### Observation endpoints

The primary observational endpoint was long-term overall survival (OS), and the secondary endpoints were progression-free survival (PFS), treatment failure mode, and adverse events of grade ≥ 3. Cox regression model was used for the multivariate analysis to evaluate the benefit value of SIB-IMRT.

### Statistical analysis and follow-up

All statistical analyses were performed using the SPSS 22.0 software package (IMB Corp., Armonk, NY, USA). OS and PFS was assessed using the Kaplan–Meier method, and difference between the groups was assessed using the log-rank test. The patients who were lost to follow-up were calculated according to survival at the last follow-up. A Cox regression was used to analyze the prognostic factors. The case–control selection was according to the 1:1 principle, and cases were selected from the SD-IMRT group and matched with the SIB-IMRT group using the propensity score matching (PSM) module in the SPSS 22.0 software package (the biostatistical method of logistic regression was used), the matching variables included gender, age, tumor location, clinical T stage, N stage, TNM stage, GTV volume, and chemoradiotherapy combination mode of the two groups. The adopted caliper width was 0.02 and *P* < 0.05 was considered statistically significant.

## Results

### Patient characteristics

There were 109 cases in the SIB-IMRT group and 266 cases in the SD-IMRT group. Before the PSM, the baseline characteristics of the two groups were different (Table 1). In the SIB-IMRT group, there were more women and more cervical and upper thoracic cancers (*P* = 0.038, < 0.001, respectively). A total of 69 pairs (138 cases) of patients were successfully matched after PSM, numbers of patients treated with SD-IMRT or SIB-IMRT year by year are shown in the Supplementary Table. The patient characteristics of the two groups are shown in Table 1, and there was no significant difference between the two groups.

### Adverse events

The main treatment-related adverse events of the two groups were acute radiation esophagitis, acute pneumonitis, and hematological toxicity. Details of the toxic effects are shown in Table 2. Among them, 3 patients experienced grade 4 to 5 toxic effects (2.2%), including one case of grade 4 radiation esophagitis (stenosis, pain, severely affecting eating and life, and the patient received gastrointestinal nutrition tube implantation, intravenous anti-inflammatory, acid inhibition, mucosal protection, and nutritional support treatment), and two cases of grade 5 radiation pneumonitis (death 1 month after radiotherapy due to respiratory failure secondary to pulmonary infection), all in the SD-IMRT group. Grade 3 adverse events occurred in 14 patients (10.1%), including radiation esophagitis in four cases (SD-IMRT group, three cases [4.3%]; SIB-IMRT group, one case [1.4%]); radiation pneumonitis in five cases (SD-IMRT group, three cases [4.3%]; SIB-IMRT group, two cases [2.9%]); hematological toxicity in five cases (SD-IMRT group, one case [1.4%]; SIB-IMRT group, four cases [5.8%]). Due to this being a retrospective analysis, the study failed to obtain the occurrence of late adverse reactions in the two IMRT mode groups.

**Table 2:** Toxic events among 138 patients given SD-IMRT and SIB-IMRT

AEs	SD-IMRT					SIB-IMRT					χ^2^	*P*-value
	Grade 0	Grade 1	Grade 2	Grade 3	Grade 4 or 5	Grade 0	Grade 1	Grade 2	Grade 3	Grade 4 or 5		
Radiation esophagitis	16	15	34	3	1	21	15	32	1	0	2.632	0.662
Radiation pneumonitis	49	14	1	3	2	54	5	8	2	0	11.789	0.009
Hematological toxicity	29	28	11	1	0	19	27	19	4	0	5.844	0.116
Nausea	46	19	4	0	0	40	24	5	0	0	1.153	0.624
Vomit	59	6	4	0	0	52	12	5	0	0	2.547	0.301
Diarrhea/constipation	64	4	1	0	0	66	3	0	0	0	1.161	0.718

### Survival and treatment failure mode

**Fig. 3:**
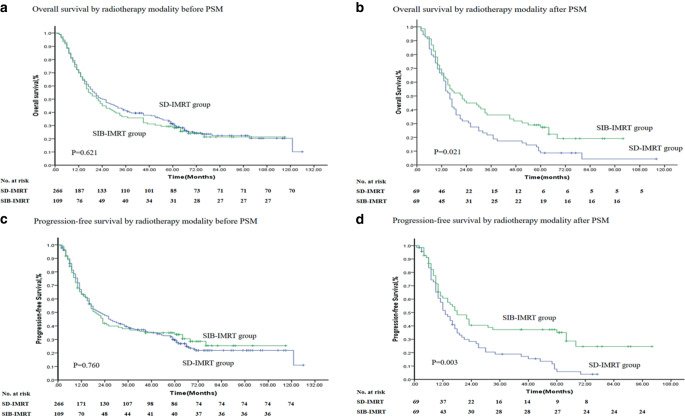
Overall survival and progression-free survival of the study population. **a** Overall survival by radiotherapy modality before PSM; **b** overall survival by radiotherapy modality after PSM; **c** progression-free survival by radiotherapy modality before PSM; **d** progression-free survival by radiotherapy modality after PSM

Until the date of follow-up, a total of 10 cases were lost to follow-up, with a follow-up rate of 97.3%. Follow-up methods included telephone calls, letters, a hospital review, and visits. According to the Kaplan–Meier method, before case-control matching, the median PFS of the SD-IMRT and SIB-IMRT groups was 22 and 19 months, respectively, with χ^2^ = 0.093 and *P* = 0.760. The 1‑, 3‑, and 5‑year overall survival rates of the two groups were 70.3, 41.3, and 31.2% and 69.7, 36.7, and 28.3%, respectively. The median overall survival time was 24 and 22 months, respectively, with χ^2^ = 0.244 and *P* = 0.621. There was no significant difference in the PFS and OS between the two groups (Fig. 3a, c). After case matching, the median PFS of the two groups was 13 and 18 months, with χ^2^ = 8.776 and *P* = 0.003. The 1‑, 3‑, and 5‑year overall survival rates of the two groups were 66.7, 21.7, and 8.7% and 65.2, 36.2, and 27.3%, respectively. The median OS time was 16 and 22 months with χ^2^ = 5.362 and *P* = 0.021. The PFS and OS of the SIB-IMRT group were significantly better than those of the SD-IMRT group (Fig. 3b, d).

**Fig. 4:**
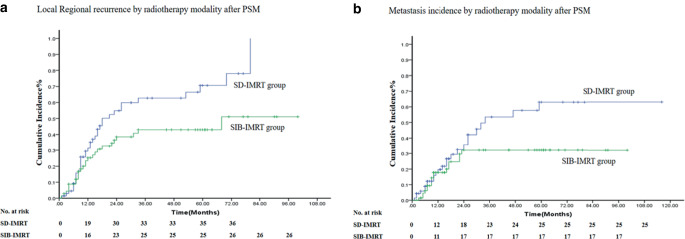
Locoregional recurrence rate and metastasis incidence of the study population. **a** Locoregional recurrence by radiotherapy modality after PSM; **b** metastasis incidence by radiotherapy modality after PSM

A treatment failure mode analysis was performed for the matched cohort, and the cumulative locoregional recurrence (primary tumor recurrence and regional lymph node metastasis) and distant metastasis of the two groups are shown in Fig. 4. The 5‑year local regional recurrence rates of SD-IMRT and SIB-IMRT were 50.7 and 36.2%, respectively, with χ^2^ = 2.949 and *P* = 0.086. The 5‑year distant metastasis rates of the two groups were 36.2 and 24.6%, respectively, with χ^2^ = 2.190 and *P* = 0.139.

### Analysis of the prognostic factors

The Cox regression model was used to screen the prognostic factors of the matched data set. The covariates that entered the analysis included gender, age, tumor location, tumor length, T stage, N stage, GTV volume, radiotherapy mode (SD-IMRT vs. SIB-IMRT), and the combination mode of chemoradiotherapy (radiotherapy alone vs. sequential chemoradiotherapy vs. concurrent chemoradiotherapy). The final independent prognostic factors selected included gender, T staging, and the radiotherapy mode. SIB-IMRT was a survival benefit factor compared with SD-IMRT (HR = 0.606, *P* = 0.008; Table 3).

**Table 3:** Univariate and multivariate Cox analyses of patients after PSM

Characteristic	Cases	Univariate analysis				Multivariate analysis		
		MST (months)	HR	95%CI	*P*-value	HR	95%CI	*P*-value
*Sex*								
Male	86	16	1	(Reference)	(Reference)	1	(Reference)	(Reference)
Female	52	22	0.573	0.387–0.847	0.005*	0.546	0.368–0.811	0.003*
*Age (years)*								
≤ 70	105	18	1	(Reference)	(Reference)	–	–	–
> 70	33	14	1.684	1.116–2.540	0.013	–	–	–
*Tumor site*								
Cervical/upper	58	21	1	(Reference)	(Reference)	–	–	–
Middle/lower	80	15	1.320	0.910–1.913	0.143	–	–	–
*Lesion length (barium meal, cm)*								
≤ 5.5 cm	78	19	1	(Reference)	(Reference)	–	–	–
> 5.5 cm	60	15	1.364	0.947–1.964	0.096	–	–	–
*T stage*								
T_1+2_	26	30	1	(Reference)	(Reference)	1	(Reference)	(Reference)
T_3_	28	15	2.420	1.319–4.441	0.004*	2.830	0.528–5.241	0.001*
T_4_	84	16	1.709	1.018–2.870	0.043*	1.800	1.070–3.027	0.027*
*N stage*								
N_0_	12	45	1	(Reference)	(Reference)	–	–	–
*N* _+_	126	16	2.242	1.042–4.823	0.039	–	–	–
*GTV volume*								
≤ 45 cm^3^	68	22	1	(Reference)	(Reference)	–	–	–
> 45 cm^3^	70	14	1.462	1.015–2.106	0.041	–	–	–
*Radiotherapy modality*								
SD-IMRT	69	16	1	(Reference)	(Reference)	1	(Reference)	(Reference)
SIB-IMRT	69	22	0.655	0.454–0.946	0.024*	0.606	0.417–0.878	0.008*
*Treatment regimen*								
RT alone	67	17	1	(Reference)	(Reference)	–	–	–
Sequential chemoradiotherapy	29	18	0.731	0.444–1.204	0.218	–	–	–
CCRT	42	16	1.071	0.709–1.617	0.745	–	–	–

*Statistically significant *p*-value

## Discussion

Radiation at a dose of 50 Gy combined with concurrent chemotherapy is the standard therapy for patients with localized carcinoma of the esophagus who are selected for nonsurgical treatment based on the intergroup trial RTOG 8501 [19, 20]. However, the dose of 50 Gy is relatively low compared with radiation doses used in curative CRT schemes for other carcinomas such as lung cancer and head and neck cancer, and higher locoregional control rates are achieved in these tumors [21, 22]. In an attempt to improve locoregional control, the randomized RTOG INT 0123 trial [23] compared CRT using a high dose (64.8 Gy/1.8 Gy) with a standard dose (SD, 50.4 Gy/1.8 Gy) combined with concurrent chemotherapy. There was no significant difference in locoregional failure (52 vs. 56%) or in the 2‑year overall survival (OS; 31% vs. 40%) between the high- and standard-dose arms. Since then, radiation of 50.0–50.4 Gy has come to be considered as the standard dose of definitive chemoradiation (dCRT) for esophageal cancer. After entering the era of precision radiotherapy, a recent randomized controlled study (ARTDECO) [14] also showed that in dCRT for esophageal cancer, a radiation dose escalation up to 61.6 Gy to the primary tumor did not result in a significant increase in local control over 50.4 Gy, and the absence of a dose effect was observed in both adenocarcinoma (AC) and squamous cell carcinoma (SCC). Hence, most centers are actually de-escalating the radiation dose worldwide.

However, for esophageal cancer, local control is always the key to success of treatment, especially for patients undergoing nonsurgical treatment. In the RTOG 8501 trial, the locoregional failure rate after dCRT was high (47%), and this was also demonstrated in several other large dCRT series [23–26]. Based on the results of a meta-analysis [27], a prescription dose ≥ 60 Gy was found to be more conducive to improving the overall survival and local control of esophageal squamous cell carcinoma in Asian countries. In a retrospective analysis of our center [28], high-dose concurrent chemoradiotherapy (cCRT) of 60 Gy produced long-term OS and LC benefits compared with the standard-dose cCRT of 50.4–54.0 Gy, with 10-year OS rates of 24 and 13.3%, respectively, and 10-year LC rates of 52.0 and 29.8%, respectively. Hence, in the guidelines of the Chinese Society of Clinical Oncology (CSCO), 50–60 Gy of radiation is the standard dose for dCRT.

As an advantageous radiotherapy technology, SIB-IMRT has been widely used in the treatment of esophageal cancer in recent years. Based on the research results of several phase I/II phase clinical trials [8–11], it is safe and feasible to use SIB-IMRT technology to increase the total dose to a primary tumor to 59.92–70.00 Gy with a single-fraction dose of 2.14–2.80 Gy. However, whether this technology can provide final local control or a survival benefit to the treatment of esophageal cancer is still questionable due to a lack of sufficient research data [12–14]. Based on the aforementioned research, this study retrospectively analyzed a large number of patients with esophageal cancer treated in a single center of our hospital. For the SIB-IMRT group, a lower dose (50.4–60 Gy) was delivered to the PTV_-C_, and the dose delivered to the PTV_-G_ was escalated to a higher level (59.92–66.00 Gy). The purpose was to explore whether dose escalation with SIB-IMRT technology might be beneficial in certain esophageal cancer patients. After screening according to the inclusion and exclusion criteria, 375 eligible patients entered the final analysis. It was observed that there were significant differences in the baseline characteristics between the two groups before PSM. The SIB-IMRT group had more women and more cervical and upper thoracic cancers (*P* = 0.038, < 0.001, respectively). Before case matching, the median PFS of the SD-IMRT and SIB-IMRT groups was 22 and 19 months, respectively, and the median overall survival time was 24 and 22 months, respectively. There was no significant difference in the PFS and OS between the two groups. After case matching, it was found that both in terms of PFS and OS, SIB-IMRT had significant advantages. Compared with SD-IMRT, the benefit time of PFS was extended by 5 months, and the benefit time of OS was extended by 6 months. This suggested that SIB-IMRT provided survival benefits for locally advanced esophageal cancer. Further observation of the treatment failure mode of the two groups showed that fewer patients in the SIB group experienced locoregional recurrence and distant metastasis: the SIB-IMRT technique tended to show a benefit. It was speculated that the local dose escalation advantage of SIB-IMRT improved the locoregional control of patients, and this then transformed into a long-term survival benefit. The results based on the multivariate analysis also showed that compared with SD-IMRT, SIB-IMRT was an independent prognostic factor for long-term patient survival and reduced the patient’s risk of death by 39.4%. Based on the above analysis, it can be considered that SIB-IMRT has potential locoregional control and survival benefits for ESCC.

In terms of treatment safety and adverse events, there were no grade 4–5 adverse events in the SIB-IMRT group: the total incidence of grade 3 adverse events was 10.1% (7/69), of which the incidence of grade 3 radiation esophagitis was 1.4% (1/69). The incidence of grade 3 acute radiation pneumonitis was 2.9% (2/69), which was similar to the clinically reported data and also showed the safety of SIB-IMRT technology. In the control group there were three cases of grade 4–5 toxicity events, and these were considered to be related to the higher radiation dose exposure of PTV_-C_ (the prescribed dose of PTV_-C_ in the SD-IMRT group was 60 Gy, and that in the SIB-IMRT group was 50.4–60.0 Gy).

This study has the following limitations: 1) the study enrollment timespan was large (2003–2014), and there might have existed large differences in technical equipment and treatment factors; 2) although the PSM method was used to balance the differences in the baseline characteristics, and there was no significant difference between the two groups, we could observe that several factors numerically favored the SIB-IMRT group after PSM, e.g., more smaller lesions, fewer patients who received RT alone, etc., which might have skewed the final outcome analysis; 3) the observation and recording of treatment-related toxicity may not be sufficiently detailed and accurate, and the late toxicity could not be obtained. Therefore, the conclusions of this study still require further confirmation in prospective studies.

Based on the above results, it was considered that SIB-IMRT was safe and reliable compared with SD-IMRT. In addition, SIB-IMRT had locoregional control advantages and potential survival benefits.

## Supplementary Information

Supplementary Table

## References

[1] Franceschini D, Paiar F, Meattini I (2013). Simultaneous integrated boost-intensity modulated radiotherapy in head and neck cancer. Laryngoscope.

[2] Leclerc M, Maingon P, Hamoir M (2013). A dose escalation study with intensity modulated radiation therapy (IMRT) in T2N0, T2N1, T3N0 squamous cell carcinomas (SCC) of the oropharynx, larynx and hypopharynx using a simultaneous integrated boost (SIB) approach. Radiother Oncol.

[3] Jin X, Yi J, Zhou Y (2014). A new plan quality index for nasopharyngeal cancer SIB IMRT. Phys Med.

[4] Yi J, Huang X, Gao L (2014). Intensity-modulated radiotherapy with simultaneous integrated boost for locoregionally advanced nasopharyngeal carcinoma. Radiat Oncol.

[5] Hakariya T, Obata S, Igawa T (2014). Feasibility of simultaneous integrated boost IMRT (SIB-IMRT) for castrationresistant prostate cancer. Anticancer Res.

[6] Franco P, Arcadipane F, Ragona R (2016). Locally advanced (T3-T4 or N) anal cancer treated with simultaneous integrated boost radiotherapy and concurrent chemotherapy. Anticancer Res.

[7] Tomasoa NB, Meulendijks D, Nijkamp J (2016). Clinical outcome in patients treated with simultaneous integrated boost-intensity modulated radiation therapy (SIB-IMRT) with and without concurrent chemotherapy for squamous cell carcinoma of the anal canal. Acta Oncol.

[8] Li C, Ni W, Wang X (2019). A phase I/II radiation dose escalation trial using simultaneous integrated boost technique with elective nodal irradiation and concurrent chemotherapy for unresectable esophageal cancer. Radiat Oncol.

[9] Sakanaka K, Ishida Y, Fujii K (2021). Radiation dose-escalated chemoradiotherapy using simultaneous integrated boost intensity-modulated radiotherapy for locally advanced unresectable thoracic esophageal squamous cell carcinoma: a single-institution phase I study. Clin Oncol (R Coll Radiol).

[10] Chen DW, Menon H, Verma V (2019). Results of a phase 1/2 trial of chemoradiotherapy with simultaneous integrated boost of radiotherapy dose in unresectable locally advanced esophageal cancer. JAMA Oncol.

[11] Yu W, Cai YW, Liu Q (2015). Safety of dose escalation by simultaneous integrated boosting radiation dose within the primary tumor guided by (18)FDG-PET/CT for esophageal cancer. Radiother Oncol.

[12] Yu WW, Zhu ZF, Fu XL (2014). Simultaneous integrated boost intensity modulated radiotherapy in esophageal carcinoma:early results of a phase II study. Strahlenther Onkol.

[13] Xu YJ, Wang C, Liu G (2016). The efficacy and safety of simultaneous integrated boost intensity-modulated radiation therapy for esophageal squamous cell carcinoma in Chinese population: a single institution experience. J Cancer Res Ther.

[14] Hulshof MCCM, Geijsen ED, Rozema T (2021). Randomized study on dose escalation in definitive chemoradiation for patients with locally advanced esophageal cancer (ARTDECO study). J Clin Oncol.

[15] Ilson DH, Forastiere A, Arquette M (2000). A phase II trial of paclitaxel and cisplatin in patients with advanced carcinoma of the esophagus. Cancer J.

[16] Petrash S, Welt A, Reinacher A (1998). Chemotherapy with cisplatin and paclitaxel in patients with locally advanced, recurrent or metastatic oesophageal cancer. Br J Cancer.

[17] Ajani JA, Fodor MB, Tjulandin SA (2005). Phase II multi-institutional randomized trial of docetaxel plus cisplatin with or without fluorouracil in patients with untreated, advanced gastric, or gastroesophageal adenocarcinoma. J Clin Oncol.

[18] Kim JY, Do YR, Park KU (2010). A multi-center phase II study of docetaxel plus cisplatin as first-line therapy in patients with metastatic squamous cell esophageal cancer. Cancer Chemother Pharmacol.

[19] Cooper JS, Guo MD, Herskovic A (1999). Chemoradiotherapy of locally advanced esophageal cancer: long-term follow-up of a prospective randomized trial (RTOG 85-01). Radiation therapy oncology group. JAMA.

[20] Kumar K, Dimri R, Khurana R (2007). A randomized trial of radiotherapy compared with cisplatin chemo-radiotherapy in patients with unresectable squamous cell cancer of the esophagus. Radiother Oncol.

[21] Machtay M, Bae K, Movsas B (2012). Higher biologically effective dose of radiotherapy is associated with improved outcomes for locally advanced non-small lung carcinoma treated with chemoradiation: an analysis of the radiation therapy oncology group. Int J Radiat Oncol Biol Phys.

[22] Yamoah K, Showalter TN, Ohri N (2015). Radiation therapy intensification for solid tumors: a systematic review of randomized trials. Int J Radiat Oncol Biol Phys.

[23] Minsky BD, Pajak TF, Ginsberg RJ (2002). INT 0123 (radiation therapy oncology group 94-05) phase III trial of combined-modality therapy for esophageal cancer: high-dose versus standard-dose radiation therapy. J Clin Oncol.

[24] Versteijne E, van Laarhoven HW, van Hooft JE (2015). Definitive chemoradiation for patients with inoperable and/or unresectable esophageal cancer: locoregional recurrence pattern. Dis Esophagus.

[25] Teoh AY, Chiu PW, Yeung WK (2013). Long term survival outcome after definitive chemoradiation versus surgery in patients with resectable squamous carcinoma of the esophagus: results from a randomized controlled trial. Ann Oncol.

[26] Welsh J, Settle SH, Amini A (2012). Failure patterns in patients with esophageal cancer treated with definitive chemoradiation. Cancer.

[27] Chen Y, Zhu HP, Wang T (2017). What is the optimal radiation dose for non-operable esophageal cancer? Dissecting the evidence in a meta-analysis. Oncotarget.

[28] Ren X, Wang L, Han C (2018). Retrospective analysis of safety profile of high-dose concurrent chemoradiotherapy for patients with oesophageal squamous cell carcinoma. Radiother Oncol.

