# Supplementary material for: Comparison of efficacy and safety between simultaneous integrated boost intensity-modulated radiotherapy and standard-dose intensity-modulated radiotherapy in locally advanced esophageal squamous cell carcinoma: a retrospective study
Source: Strahlenther Onkol. 2022 Jan 14;198(9):802–11. doi: 10.1007/s00066-021-01894-y (PMC9402727; doi:10.1007/s00066-021-01894-y)
Supplement: Supplementary file 1 — Supplementary Table [file 66_2021_1894_MOESM1_ESM.docx]

**Supplementary table**

| **Numbers of patients treated with SD-IMRT or SIB-IMRT year-by-year** | | | | | | | | |
| --- | --- | --- | --- | --- | --- | --- | --- | --- |
| **Group** | **n** | **Enrollment time(year)** | | | | | | |
|  |  | **2007** | **2008** | **2009** | **2010** | **2011** | **2012** | **2013** |
| **SD-IMRT** | 69 | 5 | 15 | 13 | 23 | 12 | 0 | 1 |
| **SIB-IMRT** | 69 | 2 | 1 | 4 | 19 | 41 | 2 | 0 |
